# Misclassification of Survey Responses and Black-White Disparity in Mammography Use, Behavioral Risk Factor Surveillance System, 1995-2006

**Published:** 2011-04-15

**Authors:** Rashid Njai, Paul Z. Siegel, Jacqueline W. Miller, Youlian Liao

**Affiliations:** Community Health and Program Services Branch, Division of Adult and Community Health, National Center for Chronic Disease Prevention and Health Promotion, Centers for Disease Control and Prevention; Centers for Disease Control and Prevention, Atlanta, Georgia; Centers for Disease Control and Prevention, Atlanta, Georgia; Centers for Disease Control and Prevention, Atlanta, Georgia

## Abstract

**Introduction:**

The validity of self-reported data for mammography differ by race. We assessed the effect of racial differences in the validity of age-adjusted, self-reported mammography use estimates from the Behavioral Risk Factor Surveillance System (BRFSS) from 1995 through 2006 to determine whether misclassification (inaccurate survey question response) may have obscured actual racial disparities.

**Methods:**

We adjusted BRFSS mammography use data for age by using 2000 census estimates and for misclassification by using the following formula: *(estimated prevalence − 1 + specificity) / (sensitivity + specificity − 1)*. We used values reported in the literature for the formula (sensitivity = 0.97 for both black and white women, specificity = 0.49 and 0.62, respectively, for black and white women).

**Results:**

After adjustment for misclassification, the percentage of women aged 40 years or older in 1995 who reported receiving a mammogram during the previous 2 years was 54% among white women and 41% among black women, compared with 70% among both white and black women after adjustment for age only. In 2006, the percentage after adjustment for misclassification was 65% among white women and 59% among black women compared with 77% among white women and 78% among black women after adjustment for age only.

**Conclusion:**

Self-reported data overestimate mammography use — more so for black women than for white women. After adjustment for respondent misclassification, neither white women nor black women had attained the *Healthy People 2010* objective (≥70%) by 2006, and a disparity between white and black women emerged.

## Introduction

Behavioral Risk Factor Surveillance System (BRFSS) data show that in 1995 the percentage of both black and white women aged 40 years or older who had received a mammogram during the past 2 years was near the *Healthy People 2010* objective of 70% (objective 3-13) (1). By 1997, the BRFSS race-specific prevalence estimates had increased and remained above the *Healthy People 2010* objective through 2006 (2). Racial differences in the validity of these self-reported data can, however, result in misclassification (defined as an inaccurate answer) of survey responses that may obscure true black-white disparities in mammography use rates (3-8).

A recent meta-analysis of studies that measured the race-specific validity of survey questions about self-reported mammography use against documented sources, such as medical and billing records, found that the specificity of survey questions that measure mammography use is lower among black women than white women (4). In this article, specificity refers to the probability that a woman who does *not* have a documented mammogram actually reports she did not have a mammogram, whereas sensitivity refers to the probability that a woman who does have a documented mammogram actually reports she did have a mammogram. After adjusting race-specific mammography use rates from the 2000 National Health Interview Survey for sensitivity and specificity, Rauscher and colleagues found increases of up to 15 percentage points in the disparity between black and white women; estimates for the percentage of women who reported having had a mammogram in the past 2 years fell from 68% to 37% for black women and from 72% to 58% for white women (4).

Death rates from breast cancer remain higher for black women than for white women, even though breast cancer incidence is higher among white women (9). This observation has been referred to as the breast cancer incidence-mortality paradox (10). Mammograms are a key tool for detecting breast cancer at an early, treatable stage and thereby reducing death rates from the disease; yet black women are more likely than white women to have advanced-stage cancer at diagnosis. BRFSS data, however, consistently indicate that black women receive mammograms as frequently as white women (11).

The objectives of this study were to adjust BRFSS mammography data for misclassification and measure the extent to which this misclassification resulted in overestimates of mammography use rates and might have obscured a disparity between black and white women. We included data from a 12-year period, 1995 through 2006, and compared adjusted rates with the *Healthy People 2010* objective that at least 70% of women aged 40 years or older should have had a mammogram within the past 2 years. We also discuss how adjusting mammography use data for misclassification may help to explain part of the breast cancer incidence-mortality paradox.

## Methods

BRFSS is the largest state-based telephone survey of the civilian, noninstitutionalized adult population. Using SAS-callable SUDAAN version 9.2 (RTI International, Research Triangle Park, North Carolina) we weighted BRFSS data for probability of selection and to match the age-, race-, and sex-specific populations from annually adjusted intercensal estimates. The survey, which uses a complex sampling design, collects self-reports of health risk behaviors monthly in all 50 states, the District of Columbia, and 3 US territories: Guam, Puerto Rico, and the US Virgin Islands. Detailed descriptions of the methods, questionnaires, and other technical survey details are available from the BRFSS website (www.cdc.gov/brfss/index.htm). We used the Women's Health Module of BRFSS, which includes mammography use questions and was administered as part of the core survey each year from 1995 through 1999 and in even-numbered years since 2000. The number of black and white women aged 40 years or older who answered the mammography use questions increased from 39,025 in 1995 to 156,982 in 2006. For the years when the Women's Health Module was not included in the BRFSS core survey — 2001, 2003, and 2005 — we used the midpoint of the prevalence estimates from the previous and following years. Chi-square analysis was used to assess differences in demographic characteristics between black and white women (*P* < .001).

A meta-analysis of 12 studies including more than 4,000 white women and 1,000 black women reported that the sensitivity of self-reported mammography questions for use within the previous 2 years was 0.97 for both black and white US women aged 40 years or older. Specificity, however, was lower for black women (0.49) than for white women (0.62) (4). These studies compared survey data of black and white women's self-reported mammography use with a review of medical or billing records to confirm whether the self-report was accurate (12-15). Women included in these 12 studies were aged 40 years or older, generally represented convenience samples from a range of low socioeconomic status (SES) to middle-upper SES groups, and were selected from intervention, clinic, and Medicare populations.

To understand the relationship between black and white estimates for mammography use independent of age, we first age-adjusted the weighted prevalence estimates to the 2000 US census standard population. Next, using the values of sensitivity and specificity obtained from Rauscher (4), we adjusted the age-adjusted prevalence estimates for misclassification, using the following formula (15): final-adjusted prevalence = *(estimated prevalence − 1 + specificity)* / *(sensitivity + specificity − 1).* Thus, for black women, the formula was (*estimated prevalence − 1 + 0.49)* / *(0.97 + 0.49 − 1),* and for white women it was *(estimated prevalence − 1 + 0.62)* / *(0.97 + 0.62 – 1).*

For these analyses, only non-Hispanic black and non-Hispanic white women aged 40 years or older who responded to the mammography questions in BRFSS were included. Respondents' race was based on self-report. Race is included as a characteristic of interest because it is a proxy for potentially unequal psychosocial and other environmental exposures (16). Race is subsequently used to examine the existence of an absolute disparity between white and black women following necessary statistical adjustment of race-specific prevalence estimates for mammography use. Chi-square tests were used to examine white-black differences in demographic data. In addition, age-adjusted and final-adjusted (adjusted for both age and misclassification) annual BRFSS prevalence estimates were tested by using linear regression trend analysis to determine whether time, race, and time-race interaction statistically affect the estimated prevalence of mammography use.

## Results

The distributions for all selected demographic characteristics differed by race (*P* < .001) (Table). Black women were younger, had a higher prevalence of employment and a lower prevalence of health insurance coverage, were less educated, and had lower incomes than their white counterparts.

Among women aged 40 years or older who participated in the 1995 BRFSS, 70% of both white and black women reported having had a mammogram during the previous 2 years (Figure). After adjustment for misclassification, the prevalence in 1995 fell to 54% among whites and 41% among blacks. In 2006, adjustment for misclassification resulted in a drop from 77% to 65% for white women and from 78% to 59% for black women. Based on final adjusted results, prevalence estimates for both black and white women in 1995 were substantially below the *Healthy People 2010* objective of 70%. Estimates were higher in subsequent years, but by 2006, they had not reached the 70% objective for either group.

**Figure 1:**
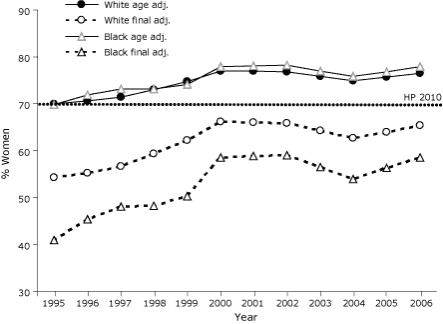
Age-adjusted and final-adjusted estimates for mammography use among white and black women, Behavioral Risk Factor Surveillance System, 1995-2006. Data refer to women who reported having a mammogram within the past 2 years. Final-adjusted estimates were obtained by adjusting the age-adjusted estimates for misclassification using the following formula with race-specific specificity (sp) and sensitivity (se) (white se = .97, sp = .62; black se = .97, sp = .49): *(estimated prevalence −* *1 + sp) / (se + sp −* *1*). See formula in Methods. Percentages for 2001, 2003, and 2005 are the averages of the previous and following years. Abbreviations: adj., adjusted; HP 2010, *Healthy People 2010* goal.

**Table:** 

**Year**	**White, %**		**Black, %**	
	**Age-Adjusted**	**Final-Adjusted**	**Age-Adjusted**	**Final-Adjusted**
1995	70.0	54.3	69.8	40.9
1996	70.6	55.2	71.9	45.4
1997	71.5	56.7	73.1	48.1
1998	73.0	59.4	73.2	48.2
1999	74.7	62.3	74.2	50.3
2000	77.0	66.1	77.9	58.6
2001	77.0	66.0	78.1	58.8
2002	76.9	65.9	78.2	59.1
2003	75.9	64.3	77.0	56.6
2004	75.0	62.6	75.8	54.0
2005	75.8	64.0	76.9	56.3
2006	76.6	65.4	77.9	58.5

The age-adjusted prevalence of mammography use among black women from 1995 through 2006 was generally equal to or slightly higher than that among white women (Figure). After adjustment for misclassification, however, prevalence estimates were on average 8.95 percentage points (*t* = −6.75, *P* < .001) lower overall among black women than among white women. Linear regression trend analysis of adjusted prevalence estimates (regressing year and race on use) also showed a significant positive relationship (β = 1.19, standard error = .19, *P* < .001) between time (years) and mammography use in the past 2 years when controlling for race. In additional trend analyses there was no significant interaction between year and race (*t* = 1.13, *P* = .27) over time (ie, the slopes of the 2 regression lines were not significantly different); thus the rate of increase in mammography use over time was the same for black and white women from 1995 through 2006.

## Discussion

According to unadjusted and age-adjusted BRFSS data, there was little to no disparity from 1995 through 2006 between the percentages of black and white women aged 40 years or older who had a mammogram during the past 2 years. Also, mammography use rates for both black and white women were consistently at or above the *Healthy People 2010* objective of 70%. However, after adjustment for respondent misclassification, mammography use rates for neither white women nor black women had attained the *Healthy People 2010* objective by 2006, and a disparity between white and black women emerged.

Lack of a disparity in mammography use between black and white women has been widely reported among studies that focus on racial, ethnic, and socioeconomic disparities (2,17,18). However, the results presented in our study suggest that previous notions of black-white parity in mammography use should be reexamined (13). Women of lower SES, immigrant women, black women, and women from other minority racial or ethnic groups remain populations of concern because of disparities in stage of breast cancer at diagnosis and breast cancer death rates (16,19-21). The disparity in mammography use observed in our study may help explain racial disparities in stage of breast cancer at diagnosis and in breast cancer death rates. Other factors that might contribute to the incidence-mortality paradox include differences in the biology of the disease between black women and white women, differences in stage at diagnosis that are unassociated with mammography use, and differences in treatment following diagnosis (11,21,22).

Lack of access to care — because of high cost, not having a usual source of care, or lack of health insurance — remains a barrier to mammography use. Lower-income, elderly, and immigrant women may encounter barriers because of language or health literacy problems. Additional factors that may reduce mammography use and contribute to disparities include patient knowledge, attitudes, and cultural beliefs (22-24). For example, Rawl and colleagues found that white women perceived greater benefits from receiving a mammogram than did black women (23).

This study has several limitations. Ideally, the measures of validity used to adjust BRFSS prevalence estimates would be based on studies of nationally representative samples of women. Although such data are not available, the studies included in the meta-analysis by Rauscher et al (4) do include white and black women from a spectrum of ages (40 years or older), regions, and SES groups. Rauscher et al do not report a significant difference between the specificity of self-reported mammography use data for black and white women (0.49 vs 0.62); however, the 95% confidence intervals for these measures do not overlap (0.42-0.57 vs 0.61-0.64). The number of studies that measured sensitivity and specificity for black women was limited; thus the values obtained by Rauscher et al may be vulnerable to sample variation, external generalizability, and other sources of measurement error. Also, the application of measures of validity from the random-effects meta-analysis does assume study-to-study variability and suggests uncertainty in estimating the underlying parameters (ie, sensitivity and specificity) (4).

Additional limitations are that the sensitivity and specificity measures that we used did not account for SES. Although sensitivity and specificity may differ between women with higher and lower SES, there is a dearth of literature in this area (4,25). The sensitivity and specificity measures also did not account for other factors such as attitudes and women's knowledge of breast cancer and screening mammography. Another limitation is that the BRFSS questionnaire (similar to other comparable national surveys) does not distinguish whether a woman received a mammogram for screening or diagnostic purposes. Finally, the median state and territorial Council of American Survey Research Organizations response rates for BRFSS have been low in recent years; from 2000 through 2006 they ranged from 49% to 58%. Median cooperation rates during the same period ranged from 53% to 77% (26).

Most studies that have measured the validity of mammography survey questions were conducted in the 1990s. These studies should be repeated to confirm whether sensitivity and specificity have changed. Such studies should be conducted in diverse populations and include an assessment of data sources such as medical and billing records.

In addition to updating validity measures of the standard wording in surveys of mammography use, it would also be useful to identify alternative wording that might have higher validity. Most surveys, including BRFSS and the National Health Information Survey, use the following introductory wording: "These next questions are about mammograms, which are X-ray tests of the breast to look for cancer." In 1992, BRFSS used different introductory wording: "I would like to ask you a few questions about a medical exam called a mammogram. A mammogram is an X-ray of the breast and involves pressing the breast between two plastic plates."

The reported prevalence of mammogram use was lower when this more graphic wording was used than when standard wording was used; this reduction in prevalence was greater among black women than white women (27). These effects on measured prevalence are consistent with the hypothesis that questionnaire language that clarifies that a mammogram involves "pressing the breast between two plastic plates" improves the specificity (ie, results in fewer false-positive responses).

Cultural sensitivity and awareness should be applied when addressing black-white and other racial/ethnic disparities in breast cancer detection and treatment. To be effective, interventions designed to overcome persistent inequalities must take into account differences in race, culture, language, SES, and age (24). Our study reinforces that these considerations can apply to the validity of surveillance data as well (4,27). Surveillance, intervention, and policy must account for the unique characteristics of women from each racial/ethnic group. Increasing mammography use — especially among underserved populations — remains a priority as public health professionals strive to eliminate breast cancer disparities and to decrease breast cancer death rates.

## Figures and Tables

**Table:** Selected Characteristics of Women Aged 40 Years or Older Who Responded to the Mammography Use Questions in the Behavioral Risk Factor Surveillance System, by Race, 1995 and 2006^a^

Characteristic	**White Women** b		**Black Women** b	

	1995, % (95% CI), n = 26,573	2006, % (95% CI), n = 119,737	1995, % (95% CI), n = 12,452	**2006, % (95% CI), n = 37,245**
**Age, y**				
40-49	32.3 (31.4-33.1)	29.5 (29.0-30.0)	38.3 (35.1-41.5)	36.9 (35.2-38.6)
50-59	21.4 (20.7-22.1)	27.9 (27.4-28.3)	22.5 (20.3-24.8)	28.3 (26.8-29.8)
60-69	21.4 (20.7-22.1)	18.6 (18.3-19.0)	21.5 (19.2-24.0)	17.6 (16.5-18.9)
≥70	25.0 (24.3-25.8)	24.0 (23.6-24.4)	17.8 (15.8-19.9)	17.2 (16.0-18.6)
**Employed**				
Yes	83.8 (82.6-84.9)	83.1 (82.6-83.7)	87.8 (84.7-90.4)	85.9 (84.2-87.5)
No	13.5 (12.5-14.6)	13.7 (13.2-14.3)	4.1 (3.0-5.7)	8.2 (7.0-9.7)
Retired	2.8 (2.4-3.2)	3.1 (2.9-3.4)	8.1 (5.8-11.1)	5.9 (5.0-6.9)
**Health insurance**				
Yes	94.2 (93.8-94.6)	93.2 (93.0-93.5)	86.6 (84.6-88.4)	85.4 (84.2-86.6)
No	5.8 (5.4-6.2)	6.8 (6.5-7.0)	13.4 (11.6-15.4)	14.6 (13.5-15.8)
**Education, y**				
<12	15.7 (15.1-16.3)	7.5 (7.2-7.8)	32.8 (30.1-35.5)	17.8 (16.5-19.2)
12	36.6 (35.7-37.4)	32.2 (31.8-32.7)	32.0 (29.3-34.8)	33.4 (31.8-35.0)
>12	47.8 (46.9-48.7)	60.3 (59.8-60.8)	35.3 (32.0-38.6)	48.8 (47.1-50.5)
**Annual household income, $**				
0-14,999	13.6 (13.1-14.2)	7.6 (7.4-7.9)	25.9 (23.5-28.5)	19.7 (18.4-21.0)
15,000-24,999	20.3 (19.6-21.0)	12.6 (12.3-12.9)	23.5 (21.2-25.9)	20.2 (18.9-21.5)
25,000-49,999	28.5 (27.7-29.3)	23.4 (23.0-23.8)	24.2 (21.1-27.7)	24.8 (23.4-26.3)
≥50,000	21.1 (20.3-21.9)	40.3 (39.8-40.8)	10.6 (9.1-12.5)	22.0 (20.5-23.5)
Not specified	16.6 (16.0-17.2)	16.1 (15.8-16.5)	15.7 (13.9-17.7)	13.4 (12.3-14.5)

Abbreviation: CI, confidence interval.

a Prevalence of all characteristics was different (*P* < .001, using *χ* ^2^ analysis) between white and black women in both 1995 and 2006.

b Unweighted sample sizes, weighted percentages.
